# Leveraging Active Learning for Failure Mode Acquisition

**DOI:** 10.3390/s23052818

**Published:** 2023-03-04

**Authors:** Amol Kulkarni, Janis Terpenny, Vittaldas Prabhu

**Affiliations:** 1 Department of Industrial and Manufacturing Engineering, The Pennsylvania State University, University Park, State College, PA 16802, USA; 2 Department of Systems Engineering and Operations Research, George Mason University, Fairfax, VA 22030, USA

**Keywords:** fault-mode acquisition, maintenance records, active learning, human-in-the-loop learning

## Abstract

Identifying failure modes is an important task to improve the design and reliability of a product and can also serve as a key input in sensor selection for predictive maintenance. Failure mode acquisition typically relies on experts or simulations which require significant computing resources. With the recent advances in Natural Language Processing (NLP), efforts have been made to automate this process. However, it is not only time consuming, but extremely challenging to obtain maintenance records that list failure modes. Unsupervised learning methods such as topic modeling, clustering, and community detection are promising approaches for automatic processing of maintenance records to identify failure modes. However, the nascent state of NLP tools combined with incompleteness and inaccuracies of typical maintenance records pose significant technical challenges. As a step towards addressing these challenges, this paper proposes a framework in which online active learning is used to identify failure modes from maintenance records. Active learning provides a semi-supervised machine learning approach, allowing for a human in the training stage of the model. The hypothesis of this paper is that the use of a human to annotate part of the data and train a machine learning model to annotate the rest is more efficient than training unsupervised learning models. Results demonstrate that the model is trained with annotating less than ten percent of the total available data. The framework is able to achieve ninety percent (90%) accuracy in the identification of failure modes in test cases with an F-1 score of 0.89. This paper also demonstrates the effectiveness of the proposed framework with both qualitative and quantitative measures.

## 1. Introduction

To improve product quality and reliability, it is necessary to implement appropriate actions to avoid faults. This is typically completed through a process known as Failure Mode and Effects Analysis (FMEA). A critical step in FMEA is to identify different failure modes, associated product subsystems, and components where faults occur. The traditional approach to improving reliability has been to perform finite element analysis to simulate and predict different failures [1,2,3,4]. Typically, failure modes are acquired through historical documents, bill of materials, and failure analysis reports. This can be a very labor intensive and time-consuming task that is both inefficient and prone to errors because of incomplete data, differences in recording maintenance information, or the lack of a standard vocabulary for maintenance records among different departments within the industry [5]. Recent advances in Natural Language Processing (NLP) are enabling automated processing of large volumes of textual data. For example, asset rich industries such as mining require substantive upfront investment specifically to acquire heavy mobile equipment. In 2022, the capital expenditure of the twenty leading miners was expected to reach USD 70.4 billion [6]. Similar situations exist in other industrial sectors such as aviation, power, and energy. Clearly, it is necessary to maintain assets over a long-life cycle to ensure return on investment along with asset maintenance data stored in company maintenance systems [7].

There are existing studies that leverage the information from maintenance logs to identify failure modes to construct a component–failure matrix using various machine learning techniques [5,8,9,10,11]. However, most of the studies focus on techniques related to a specific domain. In the cases where unsupervised learning techniques are employed, the number of clusters is typically fixed, making it necessary to provide the number of clusters or topics present within the documents. Active learning is a semi-supervised machine learning approach, which involves a human in the training stage of the model. This paper proposes to bridge this gap with the use of an online active learning method for failure mode identification. The key contributions of the paper include:

1. An uncertainty-based online active learning model for identifying and classifying faults in maintenance records;
2. Identifying some of the shortcomings in current state-of-the-art techniques related to classifying or clustering maintenance records;
3. A proposed standard vocabulary for fault modes across specific industries.

The following section provides a review of the literature including recent developments to extract failure modes using NLP along with a brief overview of the issues with maintenance texts. The next section presents the research framework and different querying techniques in online active learning. The Case Study section demonstrates the advantage of using the proposed active learning model over NLP techniques on mining equipment maintenance data. The paper concludes with a discussion of findings, benefits of the framework, and proposed future work.

## 2. Literature Review

Natural Language Processing (NLP)-based methods have been used in the past to obtain failure modes. Extraction of standard failure modes using failure mode classification from a failure–experience matrix [12] and bill of materials with recorded failure information [13] have been successfully demonstrated. A classification method using tree kernel-based support vector machine to identify bearing failures [14] based on a set category of failure modes has also been accomplished. However, these classification methods pre-suppose the type of failure modes, which is not applicable to complex systems, such as industrial robots, wind turbines, and more. Such complex systems are likely to have more failure modes than the set of failure modes under consideration. 

Unsupervised learning methods such as cluster analysis have been studied in the past during the conceptual design phase of systems. For example, “K-means” clustering combined with a row addition method based on artificially established failure modes and their frequency was studied in rotating mechanical systems [15] to develop the Component–Failure (CF) matrix. The K-means clustering algorithm has also been used on preprocessed software failure text using the representative samples as cluster labels [16]. Processes to extract failure from maintenance texts using hierarchical clustering and similar distribution-based clustering methods have been explored [17]. Researchers have also explored clustering historical FMEA data to extract failure modes and convert the structured data in FMEA sheets into an evolving tree algorithm [18]. Apart from supervised and unsupervised learning, ontology-based methods have also been proposed where the failure modes are extracted from historical data on maintenance and services [11] as well as repairing verbatim data [19]. 

While promising, the methods described above do not account for the non-uniform description of failure mode data or the lack of standard vocabulary for FMEA data. Although efforts have been made to effectively maintain and utilize a standard failure mode vocabulary [13], a systematic approach to standardize failure modes has not been established. To tackle these issues, a frequency itemset mining [5] approach was proposed to construct the CF matrix. This approach, however, is computationally expensive as frequency set mining is resource-intensive and time-consuming. Frequency itemset mining has also been known to develop associations or rules that are a product of chance. 

Most clustering algorithms, such as K-means and hierarchical agglomerative clustering algorithms, are similarity-based. These algorithms represent documents as a vector space model (VSM) [20], where a vector of length ‘V’ represents each document. The weight of a word typically calculated using TF-IDF (Term Frequency Inverse Document Frequency) represents the corresponding elements of the vector. Due to the sparsity of short texts, most of the words will have TF = 1, which means TF is ineffective in calculating the weights. This representation results in high time and space complexity, creating dimensionality issues in short texts.

More recently, the focus has been on utilizing a topic modeling approach to extract hidden failure information from maintenance records [8,21]. Topic modeling has become a widely used text clustering approach to discover abstract topics and infer hidden semantic structures from a collection of documents with techniques that can be categorized as clustering, classification, or probabilistic.

The most widely used approach in topic modeling is the Latent Dirichlet Allocation (LDA) model which has been used to improve user experience [22], summarize and provide an overview of discovered themes to recommend books [23], and analyze research trends [24]. LDA is a generative probabilistic model with three levels of hierarchy [25] for collections of discrete data such as texts. The finite mixture of topics serves as an underlying model for each collection of items, which in turn is modeled as an infinite mixture over an underlying set of probabilities. While LDA works well on long text data, it has been criticized as not being suitable for inferring topics from short texts [26], having issues with scaling [27], and ignoring co-occurrence relations [28]. These issues make it unsuitable to infer topics (failure modes) from maintenance records. It also requires that the number of topics that are specified as a parameter be supplied to the model. 

To overcome the co-occurrence limitation of LDA, a Hierarchical Latent Tree Model (HLTM) has been proposed [29]. HLTM clusters documents by treating words at the bottom level as observed binary variables and words at other levels as latent binary variables representing co-occurrence patterns. While efficient when compared to LDA, this would be unsuitable for short-text clustering. A latent topic text representation model, based on the assumption that words in the same topic follow a Gaussian distribution, was proposed to overcome the limitations of vector representation models and semantic models [30]. To discover topics, it aims to represent text as a probability distribution, causing it to perform better than LDA. However, this model is likely to perform less coherently when short texts are considered due to sparsity and lack of structure. Another proposed method that clusters texts thematically is based on Formal Concept Analysis (FCA), organizing text in the form of a concept lattice for topic modeling of Twitter data [31]. FCA also facilitates the detection of new topics based on the information coming from previous topics. However, this approach does not generalize well. There are numerous approaches that serve as an alternative to LDA, yet limitations persist. 

With the advent of deep-learning and sentence embedding techniques such as BERT (bi-directional encoder representation from Transformers), GloVe (Global Vectors for Word Representation) doc2Vec, and others, short text-classification has made significant progress in recent years. For example, a recent study [32], sought to identify misinformation pertaining to COVID-19 by performing sentiment analysis utilizing Twitter data. The study focuses on attitudes, sentiments, and how misinformation affects “E-learning”. The noise in textual data is removed using a denoising auto-encoder and an attentional feature fusion mechanism is used to combine multi-level features of ELM-AE (Extreme Learning Machine—Auto Encoder) with LSTM (Long Short-Term Memory). The authors achieved an F-1 score of 0.945, which is a higher F-1 score than other state-of-the-art approaches. A similar study [33] focused on short text classification using a feature fusion framework based on BERT. The short text defined in this case pertained to Twitter data, which have 180–200 characters. However, the short text considered focused on maintenance records which have less than 40 characters, making it much more difficult to classify.

### 2.1. Active Learning

Active learning, also referred to as “query learning” or “optimal experimental design”, is a subset of machine learning where a learning algorithm can interact with the user to label unknown data points with desired outputs. It is a type of semi-supervised machine learning algorithm. The algorithm selects a subset of samples to be labeled next from a pool of unlabeled data. The fundamental belief is that if the algorithm can choose the observations it wants to learn from, it can perform better than the traditional learning algorithms with substantially fewer observations [34]. The observations under consideration can be categorized into three subsets. If N is the total number of observations under consideration, during each iteration i, N can be broken up into three subsets:

- (N–K)_i_: observations where the label is known;
- K_i_: observations where the label is unknown;
- C_i_: A subset of Ki that is chosen to be labeled.

The decision whether to query a specific label depends on whether the gain from querying the label is greater than the cost of obtaining that information. This practice can take a few different forms depending on the budget limit and other factors, namely: 1. membership query synthesis, 2. pool-based sampling, and 3. Stream-based selective sampling.

#### 2.1.1. Membership Query Analysis

Membership Query Analysis was the first active learning scenario to be investigated [35]. In this scenario, the algorithm may request a label for any unlabeled observation in the input space, including queries generated instead of the ones sampled from an underlying distribution. For finite problem domains, query synthesis is often tractable and efficient [36]. This idea has also been extended to regression learning tasks [37] for predicting the absolute coordinates of a robot hand. 

The scenario described here is not applicable to all cases since it involves the generation of synthetic data. In this method, the active learner creates its own examples for labeling. This method is suitable for situations where it is easy to generate an example of data. For instance, some have tried to classify handwritten characters using neural networks using human oracles [38]. The queries generated by the learner often contained unrecognizable symbols and were mostly artificial hybrid characters that had no natural semantic. To overcome this limitation, an innovative and promising approach using a “robot scientist” [39] has been investigated to discover metabolic pathways in yeast saccharomyces cerevisiae (Brewer’s yeast). This approach is promising in domains where humans are not the oracles.

#### 2.1.2. Pool-Based Sampling

Most real-world datasets have many unlabeled data. In pool-based active learning [40], the algorithm is trained on a fully labeled part of the data. This helps the algorithm to determine the instances that would be most beneficial to be added to the training set in the next iteration. An informativeness measure evaluates all the data before a query or a set of queries is carried out. It is one of the most popular active learning scenarios and has been studied for various applications such as image classification and retrieval [40,41,42,43]. The downside of this algorithm is the amount of memory required.

#### 2.1.3. Stream-Based Selective Sampling

This scenario assumes that obtaining unlabeled data is inexpensive. It first selects samples from the actual distribution before deciding whether to request the oracle for labels. Stream-based selective sampling, alternatively known as sequential active learning, draws out each unlabeled instance one at a time from the data before making the decision to query. If the underlying distribution is non-uniform or unknown, the queries will be sensible, coming from the real distribution [34]. 

There are different strategies to help the algorithm arrive at a decision on whether to carry out a query. One approach is to use an informativeness measure and make a biased random decision by favoring more informative observations to be queried [44]. Another approach is to select the observations that are still ambiguous to the algorithm. A naïve strategy approach is to set a threshold for the informativeness measure and to select observations that are above the threshold.

Active learning has been increasingly used in the realm of fault diagnosis [45,46,47]. A novel probabilistic framework, based on active learning, was used as an online strategy for SHM [47]. It was found that active learning was more efficient and accurate compared to traditional machine learning algorithms. The authors used vibration data from different sensors on a Z24 bridge and simulated vibration measurements from an aircraft engine to classify faults. Similarly, an active learning method based on uncertainty and complexity was proposed for gearbox fault diagnosis [46]. The authors used empirical mode decomposition–singular value decomposition (EMD-SVD) to obtain feature vectors from sensor signals, and then used active learning combined with Random Forest to train and classify faults in a gearbox. The results from both studies showed that active learning models outperformed traditional machine learning models. While the use of sensor data to identify and classify fault modes is a novel approach, it does not deal with the classification of textual data. The authors make an assumption that sensor data is readily available to identify faults. This is not the case for most industries, which are still hesitant to invest in expensive sensors for health monitoring. Hence, this paper suggests the use of maintenance records, which are maintained by most industries and require little to no investment in additional hardware for fault mode acquisition.

To recap, although there are many approaches that have been proposed for topic modeling, LDA continues to be the most frequently used algorithm. The methods that serve as alternatives to LDA are not well-suited for short-text clustering. The next section provides details of issues with short-text clustering, specifically maintenance text, which is the focus of this paper. It will also provide a brief overview of the issues with the typical text pre-processing steps/pipeline that exist in the NLP realm. To support the discussion, a summary of the literature review is provided in Table 1 below.

## 3. Overview of Maintenance Text Issues

A typical NLP pre-processing step involves:

1. Tokenization: Tokenization is the fundamental step in NLP which is a process of segmenting texts into tokens from sentences into words and characters. It is possible to use the token occurrences of a document as a vector representation. This process converts unstructured text into structured data for analysis.
2. Removing stop words: The stop word removal process involves removing common language articles, pronouns, and prepositions such as ‘and’, ‘the’, or ‘to’ in English. This process not only frees up space but also improves processing time for any ML model. Stop words can be removed by performing a lookup operation from a predefined list. It is important to note that there is no universal list of stop words. This is true especially when it comes to technical text. The trend in the past few years has shifted from using a long list of stop words to not removing any stop words at all. Because this process decontextualizes the sentences, it is typically not suitable to perform sentiment analysis.
3. Stemming: This is the process of reducing a word to its root form. Stemming typically removes prefixes and suffixes, generating the stem of the word. It is a relatively fast and simple process. The issue with stemming is that it can generate stems that are not actual words, thus affecting the accuracy of NLP.
4. Lemmatization: The base of a word in its base form or dictionary form is called a lemma. The process of reducing words to lemma is termed lemmatization. The primary difference with stemming is that lemmatization takes context and grammar into consideration. The process itself requires access to a dictionary or a knowledge base mapping words to their lemma forms. This process is slower and computationally more expensive compared to stemming.

While helpful, these steps also cause several issues with tailoring Natural Language Processing tools to expert-driven text data [48]. The pre-processing step may cause issues such as removing asset identifiers (ABB IRB 140) or result in reversing the meaning of certain orders when stop words are removed. This is problematic. When the goal is to construct a CF matrix, it becomes imperative to identify not only the failure modes but to identify the sub-system and components where the issue occurs. Therefore, Technical Language Processing (TLP) encourages semi-supervised models with human-in-the-loop to tailor NLP tools for technical text data. One approach to address this issue is to use active learning, which needs an oracle to label the data iteratively. A text classification model can then be trained to recognize the failure modes and label them on incoming data. This requires only a small amount of input from the user. Models have demonstrated classification with sufficient accuracy [37,41,42,49,50,51]. In the case of this paper, the focus is mainly on the extraction of failure modes for the purpose of sensor selection. This makes the components in which the issue occurs of less importance.

Technical texts are typically short texts that average about four to five words per row. This makes it difficult to use certain NLP tools such as TF-IDF as it requires large volumes of text and repeating words to gain meaningful information. The vectorization of high-dimensional data results in sparsity, requiring high computing and storage capacity. This makes it challenging to determine the number of clusters, and the clusters obtained will be difficult to label, which is the goal of this paper.

There are several clustering methods that have been proposed to deal with the issue of sparsity in vector representation for short-text clustering [52,53,54,55]. To enrich the representation of short text, most of the proposed approaches mentioned above use external sources such as Wikipedia or Wordnet, which sometimes lead to inconsistency. To deal with this issue, a corpus-based topic diffusion technique has been proposed which focuses on finding words that do not appear in the text that are related to its content [56]. 

Hybrid LDA models that learn vector representation were proposed to overcome the short-text clustering limitation of LDA models [57]. In a study comparing short text classification with non-negative matrix factorization (NMF), it was shown that NMF outperforms LDA [58]. Another extension of the LDA model is the Gibbs Sampling for Dirichlet Multinomial Mixture (GSDMM) model, which extends LDA with a Dirichlet Multinomial Mixture [59]. GSDMM has been shown to perform better on short texts compared to other hybrid LDA models. GSDMM not only infers the number of topics present in a document, but it also achieves a good balance between completeness and homogeneity. GSDMM assumes that the same symmetric distribution of Dirichlet priors is given to all words, which is not realistic. To overcome the limitation of GSDMM, a method that combines Chi-square statistics with GSDMM has been proposed and shown to perform well on railway maintenance data [60]. However, the stability of the model was shown to last for a shorter duration.

In summary, it is challenging to adapt the NLP text pre-processing pipeline for maintenance data. Inconsistency in performance is one of the issues with clustering models that utilize external resources to deal with the sparsity of short texts. Hybrid LDA models outperform traditional LDA models in short-text clustering. However, hybrid LDA models do not extend to maintenance texts.

## 4. Research Framework

**Figure 1:**
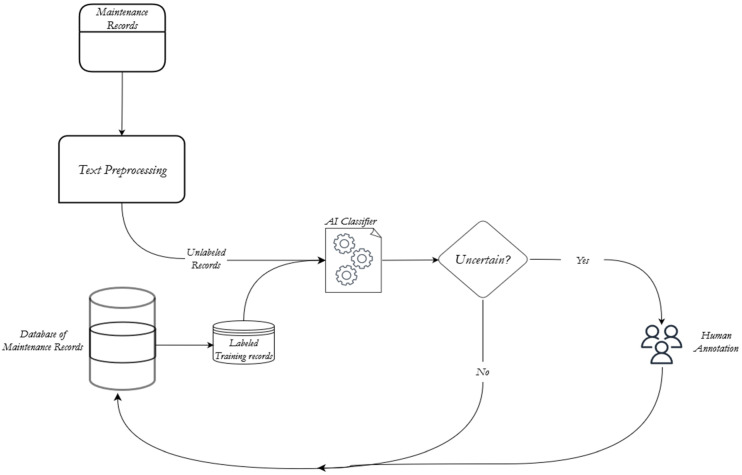
Research Framework.

Figure 1 provides an overview of the research framework. As shown, it begins with a sensor fault mapping process, which takes either the maintenance logs or FMEA data as input, whichever is available. Note that it is better to utilize maintenance logs as input if the system is deployed and operational since there is actual failure data. In case the system is still in the design phase, it is better to use FMEA data, which provides anticipated failure data.

Exploratory analysis of the raw data is conducted with the construction of word clouds, n-gram analysis, and a co-occurrence network. During the text processing stage, all special characters are removed, and any misspelled words are corrected. The pre-processed text is then clustered using non-parametric Bayesian algorithms such as GS-DMM and a hierarchical LDA model to extract the failure modes. To establish a baseline model, a similarity-based Hierarchical agglomerative clustering algorithm is used. The algorithm uses soft cosine measurement to measure the similarity between sentences. Topic modeling using LDA is also performed to illustrate its performance on short texts, specifically maintenance data. Finally, clustering using sentence embedding to identify failure modes is carried out. To support this, a supervised learning algorithm for text classification is used by an active learning model. The performance of unsupervised models is compared using Normalized Mutual Information (NMI) and Adjusted Rand Index (ARI). The supervised and active learning models are compared based on classification accuracy.

## 5. Case Study

This section demonstrates the extraction of failure modes for an operational system using maintenance records on several excavators collected from the Mobile Mining Equipment Reliability Database [61]. The maintenance logs used in this study were collected from eight excavators where the asset code has been de-identified. The data were obtained via the prognostics data library [62]. The details of how the data were collected are described by Hodkiewicz and Ho [63]. The dataset contains records of maintenance services performed on eight similarly sized excavators/assets at different mine sites across Australia over a period of ten years. The asset types have been de-identified to an uppercase letter (A, B, C, D, and E). The dataset has five variables. A brief description of each variable is provided in Table 2. Type of maintenance is a categorical variable with six levels as follows:

- PM01: unplanned or breakdown maintenance;
- PM02: planned or preventive maintenance;
- PM06: accidental damage;
- PM13: Repair of a spare for an asset;
- PM04 and PM05: unknown.

**Figure 2:**
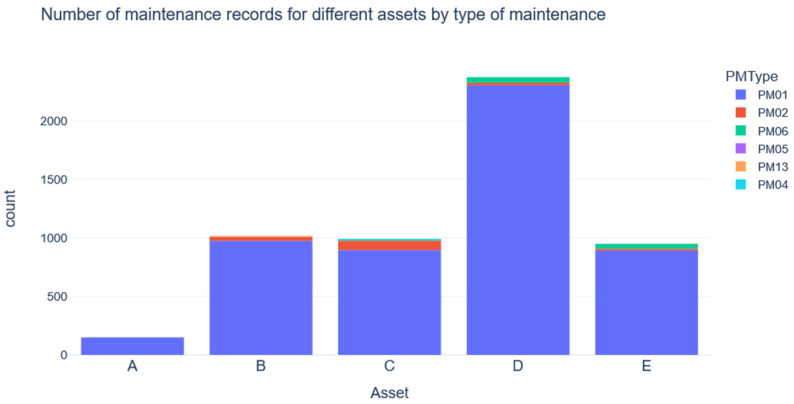
Maintenance type for different assets.

For the analysis, PM01, PM13, PM04, and PM05 are considered with a focus on identifying failure modes for sensor selection in predictive maintenance. The occurrences of each of the above-mentioned categories are shown in Figure 2. A marginal distribution plot of the cost of each asset over time is provided in Appendix A. 

**Figure 3:**
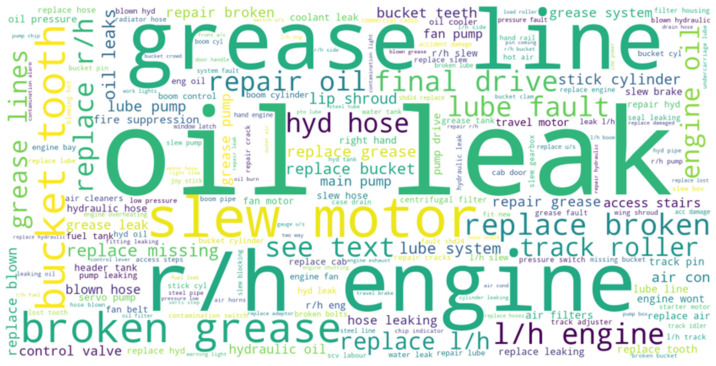
Word cloud of maintenance records.

Word clouds are powerful techniques to represent textual data. The size and color of each word indicate its frequency and importance. The word cloud generated using maintenance data (Figure 3) provides a glimpse into the dominating failures of the asset. This makes it easier to expect clusters containing certain failure modes. This process also helps in identifying the extent to which the documents need to be pre-processed.

Most of the failure modes present in Figure 3 are associated with lubricants or hydraulic fluids, indicating that lubricant leaking is a major problem in these excavators. The component that experiences frequent maintenance issues appears to be the engine on the right-hand side or ‘r/h engine’. Some of the terms are repetitive such as ‘grease line’ v/s ‘grease lines’ indicating the plural form of the line is considered a separate word. It might be possible to remove this discrepancy by stemming the word to its base form. However, doing so will make it difficult to interpret the results of the clusters to identify the cluster label or failure mode. There are some terms such as repair grease which do not create this problem. To ensure that these two terms occur together, it is necessary to conduct n-gram analysis on the data.

**Figure 4:**
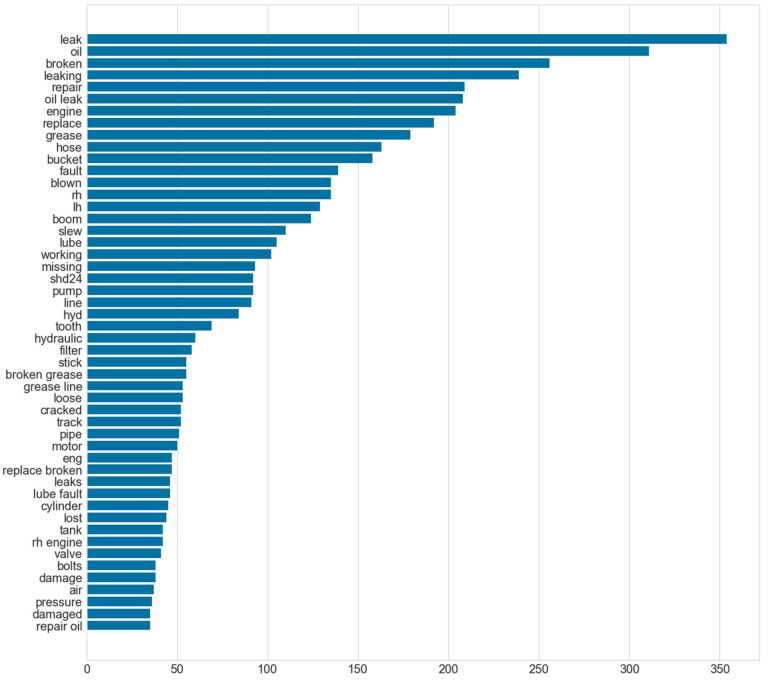
Frequently occurring words in excavator maintenance records.

**Figure 5:**
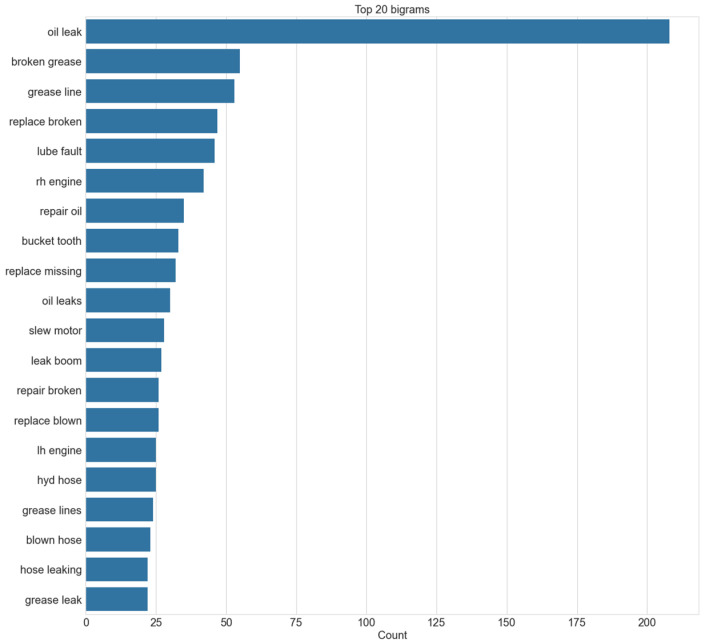
Top 20 bigrams in maintenance records.

**Figure 6:**
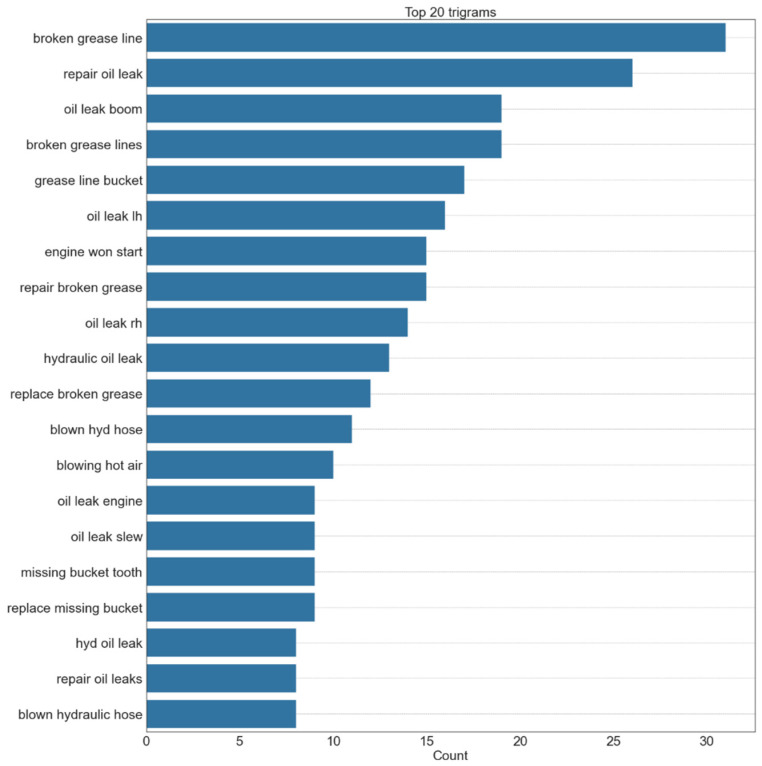
Top 20 trigrams in maintenance records.

**Figure 7:**
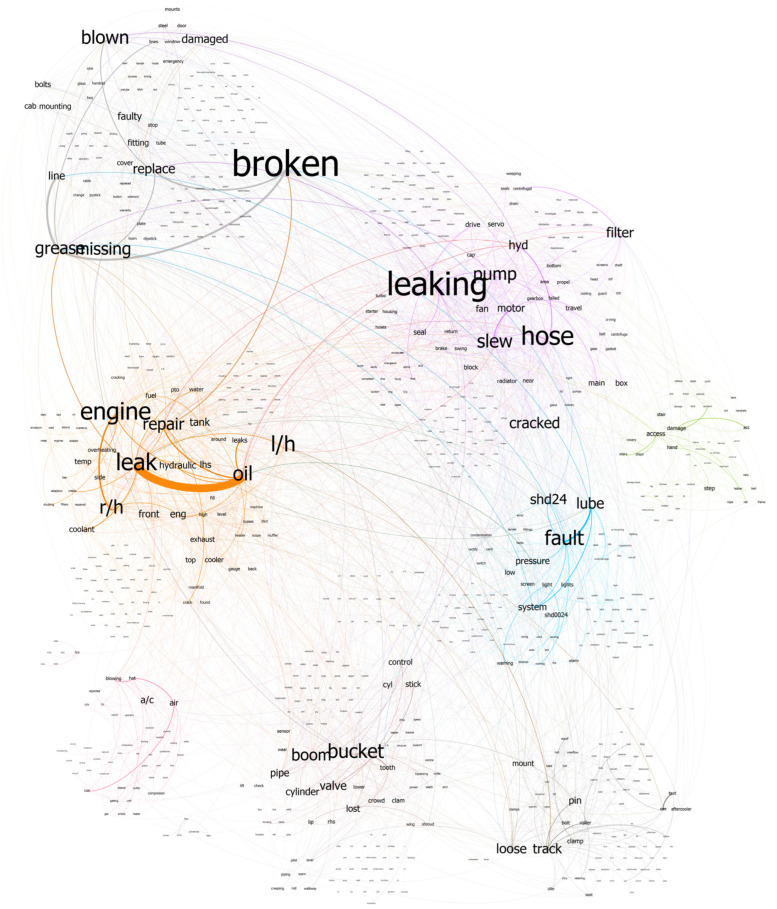
Complete co-occurrence network of corpus.

N-gram refers to a continuous sequence of n words. If the number of words is two, it is called as bigram, for three words, it is called a trigram, and so on. If the most frequently occurring words are considered in isolation, as shown in Figure 4, it would be difficult to recognize the context in which a word was used. It becomes somewhat easier to understand the texts if bigrams are considered, shown in Figure 5. However, it is still difficult to discern the failure modes for some of the entries, such as ‘rh engine’, which simply lists the name of the component where the fault is present. Considering the first step is to identify the failure modes within the document, at least three words need to be considered, shown in Figure 6. There are some repetitive observations that refer to the same failure mode but are worded differently, such as ‘repair broken grease’ and ‘broken grease line’. Some of the words refer to the same failure mode but also list the component where the fault can be observed. A co-occurrence network provides insight into which words are most likely to appear together. The higher the likelihood, the more prominent the edge between the two words. A co-occurrence network with a forced atlas 2 layout (a force-directed layout used for network spatialization) was created using Gephi, as shown in Figure 7. The size of the labels represents the degree of the associated nodes in the network. The co-occurrence network confirms most of the findings in bigrams that most of the components or lubricants need to be replaced. The major issue that these machines have is oil leaks as they are more likely to occur together. Another issue that can be inferred from this is that most of the issues appear in the ‘rh engine’ than in the ‘lh engine’.

### 5.1. Tokenization

Tokenization is a process that makes it easier to remove punctuations and numbers which typically do not provide much information in unstructured textual data. As an example, consider ‘#1 Swing Motor contamination switch’. After tokenization, it becomes ‘[‘#’, ‘1’, ‘Swing’, ‘Motor’, ‘contamination’, ‘switch’]’. It is important to note that tokenization can trigger certain complications when it comes to technical data. In the case of abbreviated words, the period following the word should be part of the same token and not a separate token. This can be problematic as technical texts often have hyphens, parentheses, and other punctuation marks. Care should be taken so that a phrase with a single meaning with white space, such as ‘New York’, is not split into two different words. 

The emerging trend seems to be to forgo tokenization entirely and develop the NLP algorithms at the character level, which makes it easier to avoid the pitfalls of tokenization. The nuances of the tokenization schemes are not enough of a crucial issue to be dealt with at the development stage of an NLP model. However, adjusting the tokenization scheme is often the easiest way to improve the model for failure modes that are worded differently, such as repair broken grease and broken grease line.

### 5.2. Text-Preprocessing

Certain words such as ‘cyl’, ‘hyd’, and ‘eng’ were changed to not count as separate words from ‘cylinder’, ‘hydraulic’, and ‘engine’, respectively. The misspelled words were corrected using TextBlob, a text processing library, and inspected manually for any incorrect corrections. Special characters were removed using regular expressions. All operations were performed in Python 3.

## 6. Results and Discussion

### 6.1. Topic Modeling

**Figure 8:**
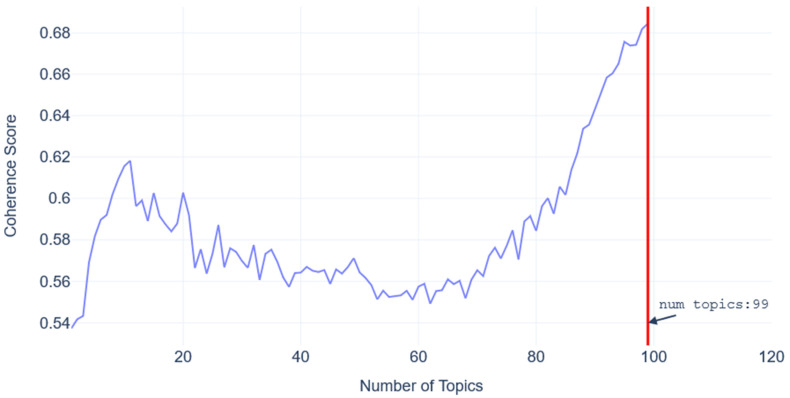
LDA coherence score as a function of the number of topics.

There are several approaches to inferring the failure modes using an unsupervised learning approach. The most popular approach is LDA. The biggest challenge when attempting to model LDA is deciding the number of topics to use as a hyperparameter. A coherence score is typically used to assess the quality of the topics, and a higher coherence score is better. The LDA was modeled using genism [62] in Python, making it easier to calculate the model coherence score. Unfortunately, for short texts, the number of topics to pick is not obvious, as shown in Figure 8. Increasing the number of topics continues to increase the coherence score, thus providing little guidance to select the number of topics. Another possibility is to select the number of topics as the first peak (topic = 10) according to Figure 8. This would result in losing information on certain failure modes or mis-categorizing them. Furthermore, this makes it difficult to ascertain failure modes when selecting the sensors to detect failure modes.

**Figure 9:**
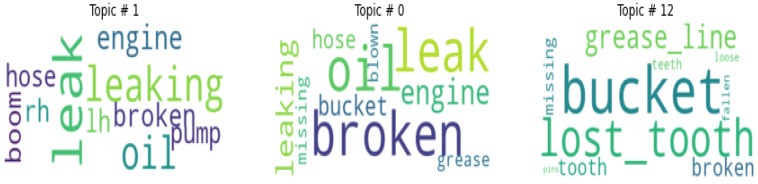
Word Cloud of Topics from HDP.

Although HDP had a lower coherence score, the number of topics discovered were closer to the ground truth. However, if the contents of the topics are plotted as a word cloud, as shown in Figure 9, it becomes evident that the topics have several of the same words that make up the bulk of the topic. This makes it difficult to identify the failure mode in each topic.

GSDMM provided the same result as HDP. Although it had a lower coherence score, the number of topics discovered was close to the ground truth. The summarized result of all three algorithms is provided in Table 3. The caveat here is that the coherence score alone is not a good statistic to indicate the performance of the model. The words that make up the topic should always be investigated before drawing a conclusion.

While some of the topics discovered made sense, many of the topics were hard to interpret or to establish a one-to-one relationship between the failure mode and the topic. This is due to the issues discussed in the section Overview of Maintenance Text Issues. Clustering with sentence embeddings is another option to extract meaningful information from short texts.

### 6.2. Clustering Texts with Sentence Embeddings

To cluster text data, it is necessary to represent them numerically. This is achieved using either embeddings or vector representations. There are several sentence embedding models that have been made available for use as pre-trained models. These models are known to perform well for sentences with semantical similarities. Given the size of the dataset, it is best to use a pre-trained model. Three different BERT models were used (all-mpnet-base-v2, all-MiniLM-L6-v2, and all-distilroberta-v1). 

The embedded sentences have more than seven hundred dimensions, making them computationally expensive. Hence, dimensionality reduction was performed using Uniform Manifold Approximation and Projection for Dimension Reduction (UMAP) [63]. Another issue with high dimensional data is the meaning of measures such as Euclidean distance and Manhattan distance in high dimensional space. 

HDBSCAN was the clustering algorithm used due to its robustness on variable density clusters. The model itself does not require the number of clusters as the hyperparameters. The clustering algorithm was used on all three sentence embedding models. However, the results were far from satisfactory. All three embedding models suggested more than fifty clusters, which is far from the ground truth. The comparison of clustering using the three different embedding models is shown in Table 4.

There can be issues with the performance of embedding models, which are known to perform well on short texts, due to the data used for training. For example, sentence embedding models use everyday language for training. This is not suitable for this case as most of the words used in the maintenance records are not everyday words. This suggests a need to train a custom embedding model using only maintenance data. While this has been accomplished [64], unfortunately, the model is not available to the public. This is most likely due to the confidentiality associated with the data.

### 6.3. Active Learning with Ensemble Modeling

In this approach, multiple models are combined in the prediction process. This is a typical solution when the following challenges are encountered during modeling:

- High Variance: The model is very sensitive to the inputs provided.
- Low Accuracy: One model fit of the entire training data may not provide accurate results.
- Noise and Bias: The model relies heavily on one or a few features for prediction.

It is challenging to create a model that would produce highly accurate results given the limitations of machine learning. Multiple models are combined to boost the overall accuracy. Reducing model error and maintaining generalization objectives can be achieved with aggregating the output from the models. There are several ensemble models such as Adaptive Boosting (AdaBoost), Random Forest, and XGBoost, to name a few. 

A random forest classifier is chosen to perform the classification task. Random Forest uses a subset of training samples as well as subset of features to build multiple trees. The goal is not to select and compare the performance of different ensemble models but to demonstrate the effectiveness of active learning. 

Different sampling methods were used to test the active learning approach using the modAL library [64] in Python. Uncertainty sampling provided the highest accuracy. The results were achieved with default parameters without any hyperparameter optimization. The model was trained on 185 out of 2303 observations. This approach improved the accuracy by 10%. It took 52 h to manually identify all failure modes present within the maintenance records. However, the active learning model took less than 10 min to identify all failure modes. This saves substantial time and cost in terms of labor hours required to identify the failure modes. 

### 6.4. Model Comparison

**Figure 10:**
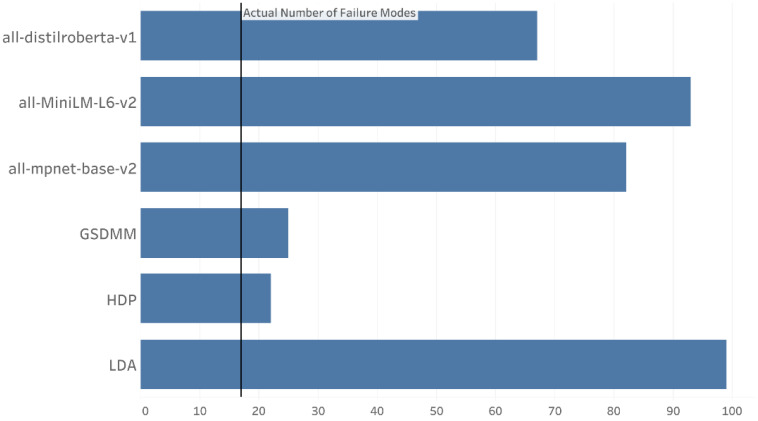
Number of Topics Discovered.

This section compares the active learning model with both unsupervised and supervised learning models that have been introduced earlier in this paper. The comparison with unsupervised learning models is shown in Figure 10. While the standard algorithm for topic modeling LDA overestimates the number of topics present in the document, both HDP and GSDMM, which are meant for short texts (<180 characters), are much closer in their estimation of the actual number of failure modes present. However, the pre-trained embedding models perform similarly to LDA, indicating that these models are not meant for short texts, especially technical texts. It is important to note that short texts are typically defined in terms of characters present in a tweet, which is not the same for technical texts (<50 characters).

An ensemble learning model, namely random forest, was the core of the active learning model. Instead of accuracy, the F-1 score is a more accurate metric for classification models with imbalanced classes. The F-1 score for the random forest model was found to be 0.86. The F-1 scores of different supervised learning models and active learning models is presented in Table 5.

It is evident from the comparisons with different models that active learning models perform better when compared to supervised or unsupervised learning models. 

## 7. Conclusions and Future Work

In this paper, a framework to extract failure modes from maintenance records using online active learning is proposed. This work illustrates the challenges faced in identifying failure modes from maintenance records. The limitations of current unsupervised learning models to perform a cluster then label approach are illustrated with a case study. These are primarily due to the limitations of the NLP tools, such as vectorization and sentence embeddings, being geared towards non-technical terms. The case study is also used to demonstrate the effectiveness of using a human-in-the-loop approach with the same NLP tools. 

While contributions made by this work are significant, there remain research avenues that can be explored to further improve the performance of both unsupervised and semi-supervised methods. An example would be training and embedding a model specifically with maintenance data. However, this requires large amounts of data, most of which are confidential, making partnerships with industry necessary. An additional area for further development using the output from this framework is to map the sensors to failure modes. In turn, this would serve as an input for sensor selection and sensor specification selection models.

## Figures and Tables

**Table 1:** Summary of Literature.

	Title	Year	Objective	Method
[15]	Failure Mode Analysis of Mechanical Systems at Conceptual Design Stage	2006	To determine the criticality of failure modes	K-means Clustering
[22]	A correlated topic model of science	2007	To improve user experience by using identified topics as guides	Correlation Topic Modeling
[17]	A case study of failure mode analysis with text mining methods	2007	To automate failure mode extraction	Multiple methods
[18]	Clustering and visualization of failure modes using an evolving tree	2015	To cluster and visualize failure modes in FMEA document as tree structures	Evolving Tree
[14]	A Preliminary Study of Clinical Abbreviation Disambiguation in Real Time	2015	To recognize clinical abbreviations	Word sense disambiguation
[19]	A data and ontology-driven text mining-based construction of reliability model to analyze and predict component failures	2016	To enhance the reliability estimation of automobiles using textual data	Ontology
[31]	A step forward for Topic Detection in Twitter: An FCA-based approach	2016	To improve topic detection process	Formal Concept Analysis
[11]	Knowledge management of automobile system failures through development of failure knowledge ontology from maintenance experience	2017	To enhance knowledge management of automobile system failures to aid in design and maintenance of automobiles	Ontology
[23]	Unsupervised Topic Modelling in a Book Recommender System for New Users	2017	To summarize the overview of discovered themes for book recommendation	LDA
[24]	Analyzing research trends in personal information privacy using topic modeling	2017	To analyze the research trends between 1972 and 2015 on personal information privacy to offer direction for future research	LDA
[29]	Latent tree models for hierarchical topic detection	2017	To model patterns of word co-occurrence and co-occurrence of those patterns to overcome LDA limitation	HLTM
[30]	Latent Topic Text Representation Learning on Statistical Manifolds	2018	To provide effective text representation and text measurement with latent topics	Gaussian Mixture Model
[47]	Probabilistic active learning: An online framework for structural health monitoring	2019	Fault mode Classification using sensor signals	Online Probabilistic Active Learning
[5]	A data-driven approach for constructing the component-failure mode matrix for FMEA	2020	Failure mode extraction of automobile seat module to build a Component–Failure Matrix for FMEA	Association Rule Mining
[21]	A Framework Based on K-Means Clustering and Topic Modeling for Analyzing Unstructured Manufacturing Capability Data	2020	To discover patterns in manufacturing capability corpus with clustering suppliers’ capability	K-means Clustering and Topic Modeling
[45]	An Applicable Predictive Maintenance Framework for the Absence of Run-to-Failure Data	2021	Predictive maintenance framework to identify and classify faults using sensor signals	Autoencoder and simple linear regression
[32]	Topic Modeling and Sentiment Analysis of Online Education in the COVID-19 Era Using Social Networks Based Datasets	2022	To identify misinformation related to COVID-19 as it pertains to E-Learning	Attentional Feature Fusion with ELM-AE and LSTM

**Table 2:** Different fields in the dataset.

Fields/Variables	Description	Data Type
BscStartDate	Date of commencement of maintenance work	Date
Asset	De-identified asset type	Categorical
OriginalShorttext	Description of maintenance work needed	String/Unstructured text
PM Type	Maintenance work type	Categorical
Cost	Cost in Australian dollars	Float

**Table 3:** Results of Topic Modeling Algorithms.

Model	Number of Topics Discovered	Coherence Score
HDP	22	0.35
LDA	99	0.68
GSDMM	25	0.33

**Table 4:** Performance of clustering sentence embeddings.

Model	ARI	NMI	Loss	Clusters
all-mpnet-base-v2	0.036	0.344	0.100	82
all-MiniLM-L6-v2	0.041	0.373	0.122	93
all-distilroberta-v1	0.040	0.326	0.125	67

**Table 5:** F-1 scores of supervised and active learning models.

Model	F-1 Score
Random Forest	0.86
Stochastic Gradient Descent	0.83
K-nearest neighbors	0.80
Decision Trees	0.67
ADA Boost	0.70
Support Vector Machines	0.82
Multi-Layer Perceptron	0.83
Entropy Sampling	0.85
Uncertainty Sampling	0.88
Margin Sampling	0.83

**Figure A1:**
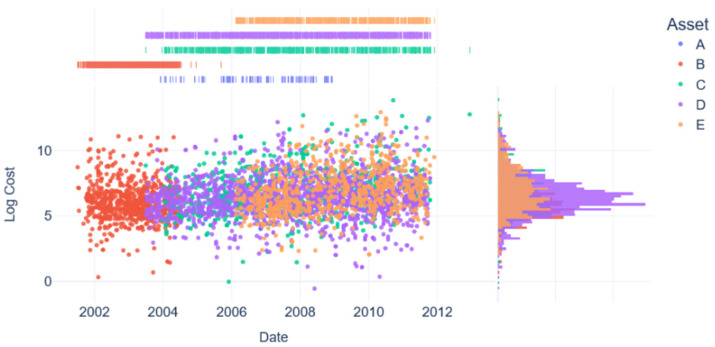
A marginal distribution plot of the cost of each asset over time.

## Data Availability

https://prognosticsdl.systemhealthlab.com/dataset/excavator-maintenance-work-orders (accessed on 17 January 2023).
